# Correction for Korgaonkar and Whiteley, “*Pseudomonas aeruginosa* Enhances Production of an Antimicrobial in Response to *N*-Acetylglucosamine and Peptidoglycan”

**DOI:** 10.1128/jb.00209-25

**Published:** 2025-09-12

**Authors:** Aishwarya K. Korgaonkar, Marvin Whiteley

## AUTHOR CORRECTION

Volume 193, no. 4, p. 909-917, 2011, https://doi.org/10.1128/jb.01175-10. Page 913: Fig. 2B and its legend should appear as shown in this correction.

**Fig 2:**
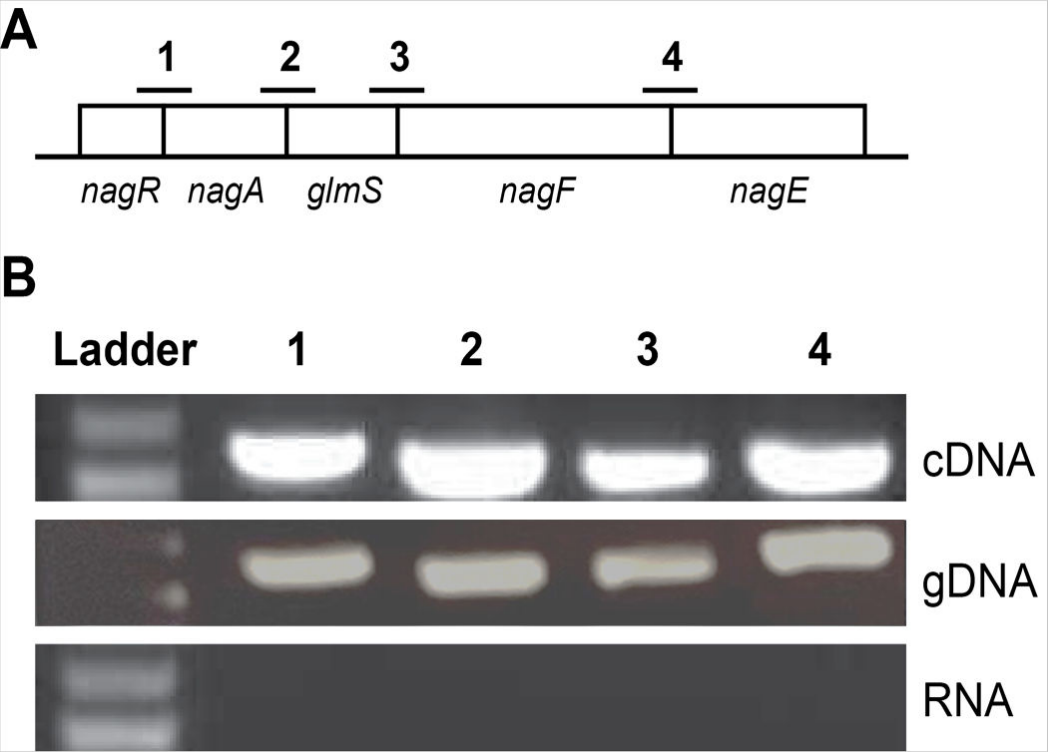
(**B**) Positive control amplicons from *P. aeruginosa* genomic DNA (gDNA) were incorrect in the original publication and have been replaced with the correct gel image form the original experiment. Due to the age and quality of the original printed images, some visual artifacts may appear as faint splice lines.

